# Chest X-ray Findings and Prognostic Factors in Survival Analysis in Peritoneal Dialysis and Hemodialysis Patients: A Retrospective Cross-Sectional Study

**DOI:** 10.3390/medicina60081331

**Published:** 2024-08-16

**Authors:** Nilgun Tan Tabakoglu, Osman Nuri Hatipoglu

**Affiliations:** 1 Hospital Health Research and Practice Center, Faculty of Medicine, Trakya University, Edirne 22030, Turkey; 2 Department of Pulmonary Diseases, Faculty of Medicine, Trakya University, Edirne 22030, Turkey; nhatip@gmail.com

**Keywords:** peritoneal dialysis, hemodialysis, etiology, mortality, survival, chest radiography

## Abstract

*Background and Objectives*: This study aims to analyze survival in peritoneal and hemodialysis patients using chest radiography and biochemical parameters, determine common dialysis etiologies and causes of death, reveal prognostic factors, and contribute to clinical practice. *Materials and Methods*: A retrospective cross-sectional study was conducted with data from 33 peritoneal dialysis and 37 hemodialysis patients collected between October 2018 and February 2020. Survival and mortality were retrospectively tracked over 70 months (October 2018–June 2024). Chest X-ray measurements (cardiothoracic index, pulmonary vascular pedicle width, right pulmonary artery diameter, diaphragmatic height) and biochemical parameters (urea, albumin, creatinine, parathormone, ferritin, hemoglobin, arterial blood gas, potassium) were analyzed for their impact on survival. Statistical analyses included descriptive statistics, chi-square test, Fisher’s exact test, Bayesian analysis, McNemar test, Kaplan–Meier survival analysis, Cox regression, Bayesian correlation test, linear regression analysis (scatter plot), and ROC analysis. SPSS 20.0 was used for data analysis, with *p* < 0.05 considered statistically significant. *Results*: Hypertension, type 2 diabetes, and urogenital disorders were the main dialysis etiologies. Peritonitis (38.5%) and cardiovascular diseases (47.4%) were the leading causes of death in peritoneal and hemodialysis patients, respectively. Significant chest X-ray differences included pulmonary vascular pedicle width and pulmonary artery diameter in hemodialysis and diaphragm height in peritoneal dialysis. Kaplan–Meier showed no survival difference between methods. Cox regression identified age, intact parathormone levels, iPTH/PVPW ratio, and clinical status as survival and mortality factors. The iPTH/PVPW ratio cut-off for mortality prediction was ≤6.8. *Conclusions*: Age, intact parathormone levels, pulmonary vascular pedicle width, and clinical status significantly impact survival in dialysis patients. Management of hypertension and diabetes, management and follow-up of urogenital disorders, infection control, patient education, and regular cardiovascular check-ups may improve survival rates. Additionally, the iPTH/PVPW ratio can predict mortality risk.

## 1. Introduction

Chronic kidney disease (CKD) is defined as kidney damage that persists for at least 3 months or that has a glomerular filtration rate (GFR) below 60 mL/min/1.73 m^2^, and the number of people diagnosed with CKD is increasing worldwide [1]. Therefore, it is a global public health problem. End-stage renal failure (ESRF) is the last stage of CKD, in which people with a GFR below 15 mL/min/1.73 m^2^ require renal replacement therapies (RRT) to sustain their lives [2]. Hemodialysis (HD), peritoneal dialysis (PD), and kidney transplantation are the most common RRT methods [3]. The prevalence of ESRD is projected to increase further in the coming years due to the increasing elderly population, longer life expectancy, and increasing prevalence of type 2 diabetes mellitus and hypertension. According to the European Renal Association—European Dialysis and Transplantation Association (ERA-EDTA) annual report published in 2020, 84,889 new patients started RRT across Europe in 2020. By the end of the same year, the total number of patients requiring this treatment reached 590,092 [4]. Another study in 2015 estimated that by 2030, the number of people who will need RRT for ESRD will reach 5.4 million worldwide [5]. Another study predicted that by 2040, CKD is projected to be the fifth most common cause of death worldwide [6]. These findings show the importance of taking preventive measures for kidney health and increasing access to RRT.

HD is the most commonly used method of RRT [7]. However, the choice of RRT modality is usually made by the physician and the patient considering various factors. Most studies have shown that HD and PD have similar survival rates [8]. Routine chest radiographs in PD and HD patients play a critical role in long-term complication management by enabling early detection of pulmonary edema, cardiomegaly, pleural effusion, and pulmonary infections. It also allows monitoring of pathologic changes in the lung parenchyma and conditions such as chronic obstructive pulmonary disease. Enabling the calculation of the cardiothoracic index (CTI) helps to identify early signs of cardiovascular complications such as heart failure and vascular calcifications. Thus, treatment strategies can be guided in a timely and accurate manner [9,10,11]. PD and HD patients are routinely followed up with various biochemical parameters to monitor the efficacy of dialysis treatment, to monitor critical clinical information such as fluid balance and nutritional status, and to prevent possible complications. These parameters include serum electrolyte levels (sodium, potassium, calcium, phosphorus), urea and creatinine levels, albumin and total protein, hemogram, ferritin, and parathormone (PTH) [12].

Understanding the risk factors in these patients is crucial for predicting survival time. Decreased renal function in HD patients leads to the accumulation of uremic toxins. Increased levels of uremic toxins impair endothelial cell function and trigger inflammation, oxidative stress, and vascular calcification processes, leading to cardiovascular diseases. Therefore, the primary cause of death in HD patients is cardiovascular disease [13]. In PD patients, infections and complications caused by infections have been accepted as the primary cause of death due to the constant connection of the peritoneal catheter with the external environment [14].

Survival analysis using chest radiography and routine biochemical parameters is critical in identifying prognostic factors in PD and HD patients. These analyses allow clinicians to develop individualized treatment strategies according to the needs of patients, initiate treatment early, and increase long-term survival rates. However, there is no study in the current literature comparing and analyzing chest radiographs and routine biochemical parameters of PD and HD patients.

Intact parathyroid hormone (iPTH) level is a critical biochemical marker in dialysis patients. iPTH plays a vital role in calcium and phosphate metabolism and contributes to the maintenance of vascular integrity [12]. Pulmonary vascular pedicle width (PVPW) is a radiographic measurement reflecting central blood volume and the state of the pulmonary circulation. PVPW may indicate fluid overload, which is a common complication in dialysis patients and can lead to adverse cardiovascular outcomes [15]. In this case, the iPTH/PVPW ratio may emerge as a new prognostic marker that integrates biochemical and radiologic parameters, providing important information about dialysis patients, especially their mortality.

Evaluation of the iPTH/PVPW ratio may allow clinicians to better assess mortality risk and improve survival in this vulnerable population. This study introduces the iPTH/PVPW ratio as an important marker and suggests that it may be a reliable predictor of mortality in dialysis patients. The rationale for considering this parameter is based on the prominent role of iPTH on vascular calcification and the utility of PVPW in reflecting fluid status and cardiovascular risk. At the same time, our study aims to fill the gap in the literature by analyzing survival in PD and HD patients with chest radiography and routine biochemical parameters and to reveal the prognostic factors in this patient group.

## 2. Materials and Methods

### 2.1. Study Design and Participants

This study is a retrospective cross-sectional study analyzing the data of 33 PD and 37 HD program patients (a total of 70 patients) who were followed and treated in our hospital between October 2018 and February 2020. The data include [chest radiography (every 6 months for HD patients; annually for PD patients), urea, albumin, creatinine, PTH, ferritin, hemoglobin, arterial blood gas, potassium, and phosphate]. The biochemical and chest radiography measurements are based on routine follow-ups conducted between October 2018 and February 2020. The same patients were retrospectively followed from October 2018 to June 2024 in terms of survival and mortality. The Charlson Comorbidity Index was used to determine the comorbidities that may affect the survival of the patients in our study [16]. Data were obtained from our hospital automation system, patients’ files, and the e-nabız information system. The dialysate calcium concentration used in HD patients was 1.25 to 1.50 mmol/L. PD patients included continuous peritoneal dialysis and ambulatory peritoneal dialysis patients.

For this study, patients who were previously diagnosed with ESRD based on clinical, radiologic, and laboratory data and Clinical Practice Guidelines for the Evaluation and Management of CKD (KDIGO); who received PD or HD treatment for at least 1 month; whose GFR was below 15 mL/min/1.73 m^2^; who had complete data for between of dates October 2018 and February 2020; who had monthly kt/v between 1.2 and 1.4 for patients receiving HD treatment and weekly kt/v between 1.7 and 2.0 for patients receiving PD treatment; and who were aged 18 years and older were included in the study [17,18]. Patients with incomplete data, patients whose kt/v did not meet the adequacy criteria for dialysis, patients with parathyroidectomy, and patients under 18 years of age were excluded from this study.

### 2.2. Lung Radiograph Measurement Calculations

**Figure 1:**
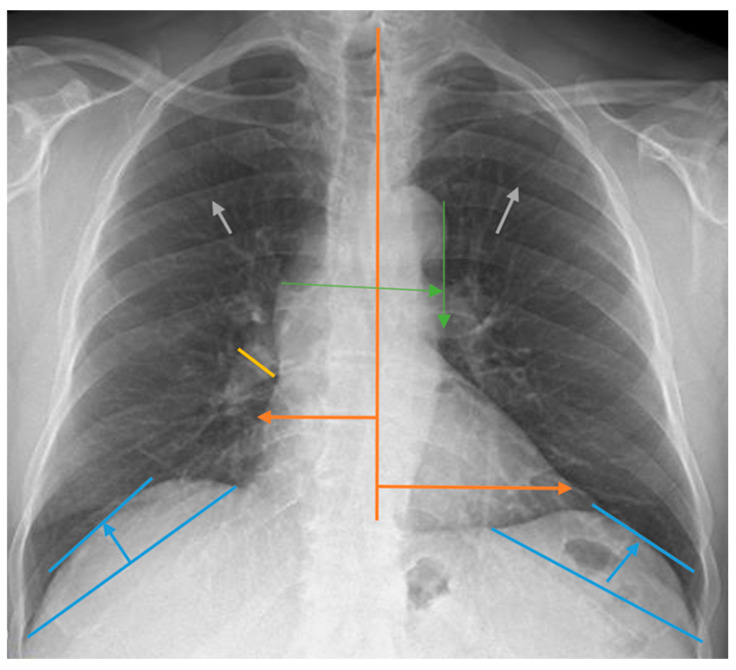
Chest radiograph measurements. Orange line: cardiothoracic index; green line: pulmonary vascular pedicle width; yellow line: right pulmonary artery diameter; blue line: diaphragmatic height; gray arrows: pulmonary redistribution.

Measurements for the evaluation of chest radiographs, CTI, heart width, pulmonary vascular pedicle width, pulmonary artery diameter, pulmonary redistribution, diaphragm height, and diaphragm width are shown in Figure 1. CTI is the ratio calculated by dividing the heart’s widest diameter by the chest cavity’s widest internal diameter. For this measurement, 0.42 and 0.50 were considered normal values. Above 0.50 was defined as cardiomegaly [19]. PVPW is used to detect heart failure and volume overload. It was measured as the distance from the outer edge of the aortic arch (just below the upper border of the left main pulmonary artery) to the outermost border of the right main pulmonary artery. PVPW < 70 mm was considered normal [15]. Right pulmonary artery diameter is used to detect pulmonary hypertension, right heart failure, and volume overload. The right pulmonary artery was measured closest to the midline of the pulmonary hilus. Right pulmonary artery diameter < 17 mm was considered normal [20]. Normal diaphragmatic height was generally considered 1.5–2.5 cm on the right and 1.0–2.0 cm on the left. This measurement is used to assess the functional status of the lungs and diaphragm. Pulmonary redistribution is the redistribution of blood flow from the lower lobes to the upper lobes of the lungs, where it is usually more concentrated. Its impairment becomes evident in diseases such as heart failure, fluid overload, and pulmonary hypertension. Other lung findings evaluated are pleural fluid, pleural thickness, alveolar infiltration, aortic calcification, and atelectasis. However, these parameters were not measured. Their presence or absence on chest radiography was taken into consideration. The acceptance criteria for radiologic status were defined as follows: improvement was indicated by a reduction in the diameter of the CTI and PVPW, a reduction in pleural fluid, and an improvement in parenchymal findings such as interstitial and alveolar changes. Stability was defined as no change in any of these parameters, while deterioration was characterized by a lack of improvement in these areas. Similarly, the criteria for clinical status in dialysis patients included improved symptoms such as fatigue and muscle cramps, enhanced laboratory results including calcium, phosphorus, serum creatinine, and urea levels, and better blood gas results. Stability was noted when there was no change in these parameters, and deterioration was identified by the absence of improvements.

### 2.3. Ethical Approval

For this study, study permission was obtained from our university’s scientific research ethics committee (TÜTF-BAEK). The Declaration of Helsinki performed all procedures in this study.

### 2.4. Statistical Analysis

Results were expressed as mean ± std. deviation, mean (95% confidence interval), or number (%). Descriptive statistics were used for the etiologic distribution of dialysis patients. The chi-square test and Fisher’s exact test were used to determine the differences in the causes of death between peritoneal dialysis and HD groups. Bayesian analysis for related samples was used to compare diaphragmatic height, pulmonary artery width, vascular pedicle width, heart size, and cardiothoracic ratio at baseline and 17 months later in PD and HD groups.

Bayesian analysis for independent samples was used to compare the differences in diaphragmatic height, pulmonary artery width, pulmonary vascular pedicle width, heart size, and cardiothoracic ratio between PD and HD groups at baseline and after 17 months. Effect size values (Cohen’s d and w) were calculated.

The McNemar test was used to compare the presence of cardiomegaly, redistribution, interstitial pattern, pleural fluid, pleural thickness, alveolar infiltration, and standard variables at baseline and 17 months in both PD and HD groups.

Kaplan–Meier survival analysis (log-rank test) was used to analyze the survival of peritoneal and HD patients. Univariate Cox regression analysis was used to examine the effects of possible factors on survival based on 2018 values. Multivariate Cox regression analysis was performed for parameters with *p* < 0.05. The correlation between iPTH level and chest radiographic findings was analyzed using the Bayesian correlation test. The relationship between iPTH and PVPW was analyzed by linear regression and shown as a scatter plot. The relationship between radiological status and clinical status was analyzed using the chi-square test. The results showed a significant correlation between radiologic status and clinical status and between iPTH and PVPW. From these correlations, backward multivariate Cox regression analysis was applied to evaluate the effect of iPTH and PVPW on mortality. In line with the significant correlation found as a result of the analysis, the power of the iPTH/PVPW ratio in predicting mortality was evaluated by the ROC curve. The SPSS 20.0 statistical package program was used for data analysis (IBM SPSS Statistics for Windows 10. Version 20.0. Armonk, NY, USA: IBM Corp.). *p* < 0.05 was accepted as the limit of statistical significance.

## 3. Results

How the chest radiograph measurement calculations used in this study were performed is shown in Figure 1.

This study included 33 PD patients and 37 HD patients. The mean age of PD patients was 52.1 ± 9.4 years, and 63.6% were female. The mean age of HD patients was 59.9 ± 16.9 years, and 62.2% were female. The distribution of etiologic causes in a total of 70 dialysis patients is shown in Table 1. Accordingly, the most common causes of dialysis were hypertension (48.6%) and type 2 diabetes mellitus (27.1%).

The distribution of causes of death in PD and HD patients is compared in Table 2.

The data revealed that the most common causes of death differed for both dialysis types. The highest cause of death in PD patients was peritonitis with 38.5%, while the highest cause of death in HD patients was other cardiovascular causes with 47.4%.

Pearson chi-square test results showed that there was a significant association between PD and HD groups in terms of causes of death (*p* = 0.030). However, since the expected frequency was less than 5 in 93.8% of the cells, Fisher’s exact test was applied. Fisher’s exact test results also confirmed that there was a statistically significant difference between dialysis types in terms of causes of death (*p* = 0.008). Fisher’s exact test results showed that the rate of death due to peritonitis was significantly higher in the PD group (*p* = 0.005), and the rate of death due to other cardiovascular causes was significantly higher in the HD group (*p* = 0.013). No significant difference was found between the groups in terms of other causes of death (Table 2, *p* > 0.05).

None of the diaphragmatic height, pulmonary artery width, pulmonary vascular pedicle width, heart size and cardiothoracic index, and biochemical parameters had a high effect size in PD and HD groups. Diaphragm height, iPTH, and potassium in the PD group, pulmonary artery width and PVPW in HD group, and also diaphragm height between PD vs. HD groups had a medium effect size. All of the other variables had a small or very small effect size in PD and HD groups (Table 3).

According to the McNemar test, there was no significant difference in the changes of cardiomegaly, redistribution, interstitial pattern, pleural fluid, pleural thickness, or alveolar infiltration variables at baseline and 17 months in both PD and HD groups (*p* > 0.05 for all; Table 4).

**Figure 2:**
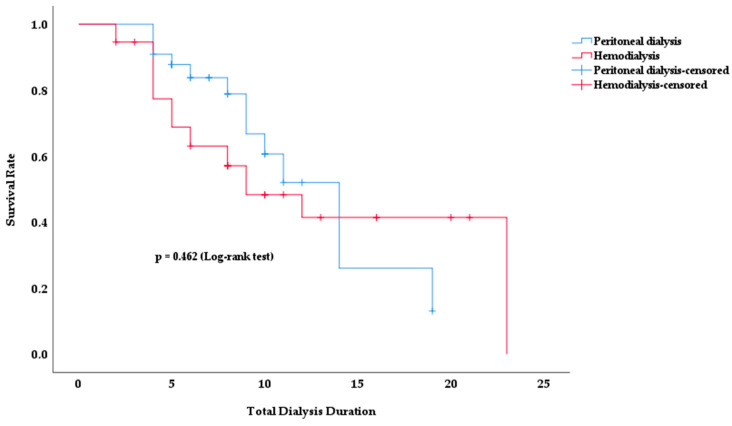
Survival rate according to total dialysis duration in PD and HD patients.

Kaplan–Meier survival analysis was used to analyze the survival rates of PD and HD patients according to total dialysis time. Figure 2 shows how the survival rates for both dialysis modalities changed over time.

No significant difference in survival rates was observed between the two dialysis modalities. Although the survival rate in the HD group appeared to be lower in earlier periods, this difference was not statistically significant (*p* = 0.462). In both groups, the survival rate decreased with increasing dialysis duration. However, no clear method of superiority emerged.

The mean survival time was 12.4 years in PD patients and 13.2 years in HD patients (*p* = 0.462). The total duration of dialysis was 9.0 ± 4.5 in survivors and 7.5 ± 4.8 in deceased patients (*p* = 0.067).

The effects of possible factors on survival were analyzed by univariate Cox regression analysis, and the results are shown in Table 5.

Univariate Cox regression analysis revealed that age, albumin, hemoglobin, ferritin, potassium, iPTH levels, and clinical status significantly affected survival in PD and HD patients, while radiologic status had a borderline significant effect on survival risk. In the Ex group, mean age (Table 5, *p* = 0.016) was higher, albumin level (Table 5, *p* = 0.005) was lower, hemoglobin level (Table 5, *p* = 0.04) was lower, and ferritin level (Table 5, *p* = 0.047) was higher. In addition, potassium level (Table 5, *p* = 0.035) was significantly lower in the Ex group, while iPTH level (Table 5, *p* = 0.013) had a statistically significant effect on survival. iPTH levels were lower in the Ex group. Clinical status had a significant effect on survival (Table 5, *p* = 0.003). This result indicates that clinical status has a positive effect on survival. Patients with radiologic deterioration had a higher survival risk than patients with stable or improving radiologic status (Table 5, *p* = 0.055), with borderline statistical significance. No statistically significant difference was found between the other parameters (*p* > 0.05 for all).

The effects of the significant parameters in univariate Cox regression analysis on survival were evaluated by multivariate Cox regression analysis; the results are shown in Table 6.

According to multivariate Cox regression analysis, the effects of age, albumin, hemoglobin, ferritin, iPTH, potassium levels, and clinical status on survival were analyzed. The effects of age, iPTH levels, and clinical status on survival were statistically significant (Table 6, *p* < 0.05). Other parameters were not significant (*p* > 0.05).

A Bayesian correlation test was performed to evaluate the correlation between iPTH level, which was found to be significant in the multivariate Cox regression analysis, and chest X-ray findings that may have an effect on survival. The results of the analysis are shown in Table 7. 

**Figure 3:**
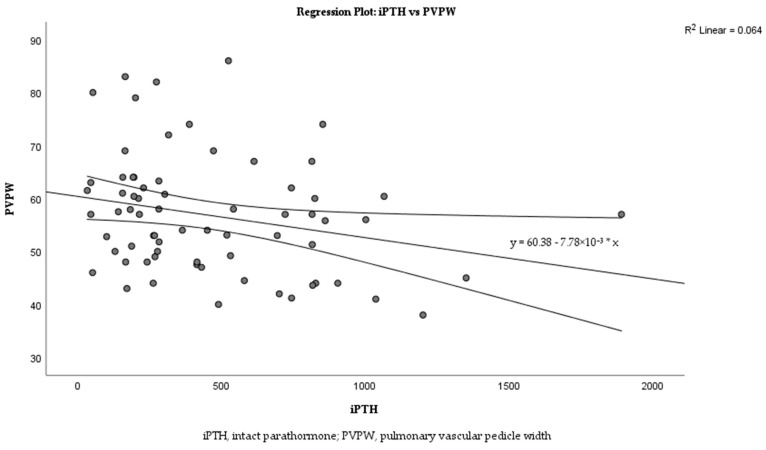
Correlation between iPTH levels and PVPW in dialysis patients.

Linear regression analysis was performed to examine the relationship between iPTH levels and PVPW in more detail. The analysis revealed a weak negative correlation between iPTH and PVPW (Figure 3; R^2^ = 0.064). The regression equation is y = 60.38 − 7.78 × 10^−3^x with a *p* = 0.036.

The relationship between radiologic status and clinical status was evaluated using the chi-square test, and the results are shown in Table 8.

These results showed a statistically significant correlation between radiologic status and clinical status (Table 8; effect size, 0.287; *p* = 0.016). On the other hand, there was a significant correlation between iPTH level and PVPW (Table 7; mean r = −0.241, 95% CI: −0.457, −0.023). No significant correlation was found between the other variables [Table 7; diaphragm height (mean r = −0.036, 95% CI: −0.265, 0.194), pulmonary artery diameter (mean r = −0.128, 95% CI: −0.352, 0.100), CTI (mean r = −0.071, 95% CI: −0.299, 0.156)]. Therefore, we calculated iPTH/PVPW and analyzed its effect on mortality by multivariate Cox regression analysis. The results are shown in Table 9.

Multivariate Cox regression analysis revealed that age, iPTH/PVPW, and clinical status had a statistically significant effect on mortality (Table 9, *p* < 0.05).

**Figure 4:**
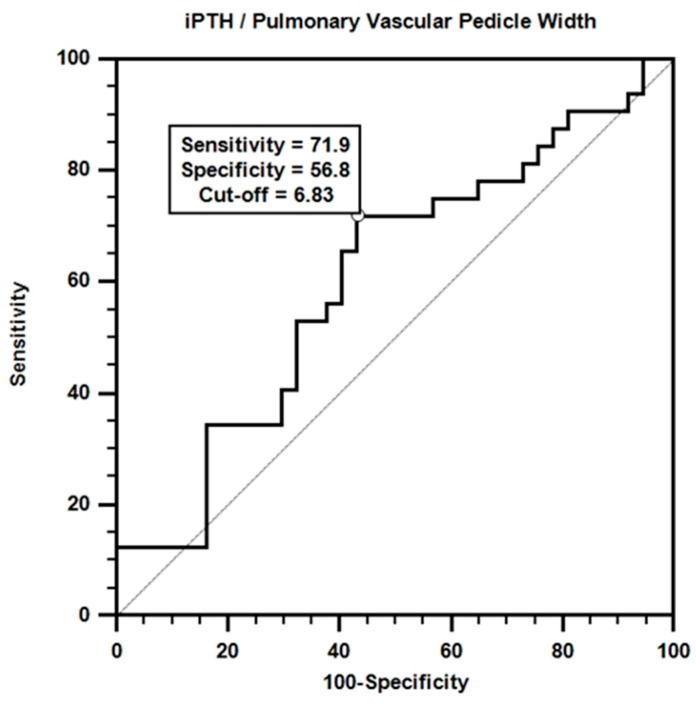
ROC curve for iPTH/pulmonary vascular pedicle width.

Receiver operating characteristic (ROC) analysis was performed to evaluate the effectiveness of iPTH/PVPW in mortality prediction. The results of the analysis are shown in Figure 4.

According to the ROC curve obtained, a certain accuracy in mortality prediction was achieved with the iPTH/PVPW combination. Although the area under the curve value was not statistically significant (AUC = 0.606; *p* > 0.05), the sensitivity value for the iPTH/PVPW ratio at the cut-off value of ≤6.8 was 71.9%, and the specificity value was 56.8%.

## 4. Discussion

In this study, we performed a retrospective comparative analysis of chest radiographs and routine biochemical parameters of PD and HD patients and analyzed these results in terms of survival in dialysis patients and prognostic parameters. Such a study is not available in the literature, and our study fills this gap. Our study revealed that there was no significant difference in survival between PD and HD patients. Furthermore, our study revealed a significant correlation between radiologic status and clinical status. Early detection and management of radiological findings such as CTI, redistribution, alveolar infiltration, interstitial pattern, pleural fluid, cardiomegaly, and aortic calcification due to vascular calcium deposition, diaphragmatic elevation, and atelectasis have been shown to affect survival positively. These results emphasize that prospective close monitoring of the health status of patients under dialysis treatment, especially the management of cardiopulmonary complications, is important in improving survival. Age, iPTH level, iPTH/PVPW, and clinical status were determined as the most important prognostic factors affecting survival and mortality. Moreover, our study suggests that the iPTH/PVPW ratio, together with clinical correlation, may play a role as a potential predictor of mortality.

In scientific and clinical applications, the main goal of prediction is to accurately estimate the risk of an outcome using a combination of several predictors. The final predictive model created in this process is based on data relationships that are often statistically significant but not necessarily causal [21]. Data selection plays a vital role in determining the survival analysis results of ESRD patients. Therefore, the use and identification of routine chest X-ray findings and routine biochemical parameters of dialysis patients is as important as causality analysis [22].

Identifying the iPTH/PVPW ratio as a novel biomarker that can be used to predict mortality risk is consistent with similar studies in the literature using omics technologies. For example, omics-based biomarkers used to predict acute rejection after kidney transplantation can be integrated into clinical practice, enabling the development of more effective strategies for patient management [23]. This demonstrates how integrating radiologic and biochemical parameters offers innovative approaches to prognosis prediction [24].

When the etiology of 70 dialysis patients was analyzed in our study, the most common causes were hypertension (48.6%), type 2 diabetes mellitus (27.1%), and urogenital disorders such as vesicourethral reflux and polycystic kidney (11.4%) (Table 1). Our findings are consistent with the existing literature. In a 2016 study, it was shown that uncontrolled hypertension may lead to progressive kidney damage and cause ESRD, which is one of the important reasons for dialysis treatment [25].

In another study, hypertension was found to be a significant contributor to the development of CKD and progression of CKD to ESRD in patients, and 90% of patients with CKD were affected by hypertension [26]. Diabetic nephropathy is a common microvascular complication of type 2 diabetes mellitus and usually progresses to ESRD, requiring dialysis. A recent study examining the etiologic distribution of CKD showed that type 2 diabetes mellitus remains one of the most important causes of ESRD [27].

Urogenital disorders have been reported to cause permanent kidney damage and contribute to dialysis patient burden by causing impairment in renal function from childhood onwards. The association of vesicourethral reflux, which is a consequence of urogenital disorders, with CKD has been investigated in a large cohort study, and it has been shown that vesicourethral reflux significantly increases the risk of developing CKD in children [28]. These results emphasize the importance of early diagnosis and treatment of hypertension, type 2 diabetes mellitus and urogenital disorders in reducing dialysis patient burden. At the same time, effective management of these diseases is essential for the survival of patients who develop ESRD, receive dialysis treatment, and have one of these chronic disease diagnoses.

In our study, peritonitis was found to be the most common cause of PD-related deaths, accounting for 38.5% (Table 2). This result is consistent with a study in which 267 PD patients were included and PD-related peritonitis was investigated [29]. The most common causes of death in HD patients were cardiovascular causes (47.4%) and myocardial infarction (10.5%) (Table 2), which is in line with the recent literature which found that 57.9% of HD patients suffered from cardiovascular diseases and almost half of HD patients died due to cardiovascular diseases [30]. Accordingly, regular patient follow-up, infection management, continuous patient education, and compliance with strict hygiene rules may contribute to improved survival rates in PD patients. In HD patients, identification, regular follow-up, and managing cardiovascular risk factors contribute to increased survival rates for this group.

When the at-17-month changes in the chest radiographs of PD patients were analyzed, it was found that diaphragmatic height, iPTH, and potassium increased significantly (Table 3; respectively, Cohen’s d = −0.62, −0.52, −0.75). In PD, the peritoneum, which is a serous membrane consisting of visceral and parietal leaves with a surface area of approximately 1–2 m^2^ in adults, is used [31]. During PD, dialysate is delivered daily through a catheter into the peritoneal cavity, which is performed on a daily basis, and due to inadequate ultrafiltration, a certain amount of fluid accumulates in the peritoneal cavity each time, resulting in volume overload [32]. Dialysate fluid introduced into the peritoneal cavity increases intra-abdominal pressure and elevates the diaphragm [33]. In addition, the reason for the increase in iPTH and potassium levels in PD patients may be excessive consumption of foods containing high potassium and phosphorus in the diets of PD patients [34]; impairment of bone mineral metabolism with the progression of chronic kidney disease and consequent increase in iPTH levels in secondary hyperparathyroidism [35]; and patients not using their medications regularly. Our findings are consistent with the existing literature.

When chest radiographs of HD patients were compared 17 months apart, it was found that PVPW and pulmonary artery diameter increased significantly (Table 3; respectively, Cohen’s d = −0.63, −0.50).

HD is usually administered two or three times a week, each treatment session lasting at least 4 h. Between these sessions, patients are expected to pay attention to fluid intake, and interdialytic weight gain should not exceed 4–4.5% of their dry weight [36]. If patients do not pay attention to fluid intake, volume overload occurs, resulting in increased pulmonary artery pressure and pulmonary vascular pedicle enlargement. Patients may develop heart failure due to chronic volume overload, which increases pulmonary artery pressure and leads to enlargement of both the pulmonary vascular pedicle and pulmonary artery diameter. Arterio-venous fistulas used in HD patients increase pulmonary artery pressure by increasing cardiac output. For all these reasons, pulmonary hypertension is also common in HD patients. And this is seen as an increase in PVPW and pulmonary artery diameter on chest radiography [37]. Our results are in parallel with these findings reported in the literature. Urea, albumin, ferritin, arterial blood gas, hemoglobin, and creatinine values of HD and PD patients at baseline and 17 months later were compared both within and between groups and found to have small or very small effect sizes in the PD and HD groups (Table 3). The main reason for this result is that both HD and PD show similar efficacy in removing metabolic wastes and maintaining electrolyte balance. Other essential factors are the optimal level of dialysis adequacy of the patients since the start of our study, implementation of current treatment protocols, and control of nutrition and drug therapy. These factors led to stable biochemical parameters and minimal changes throughout this study. Thus, no significant differences were observed.

Our study compared the chest radiographic findings of PD and HD patients. A statistically significant difference was found between the two groups in terms of diaphragmatic height (Table 3; 95% CI: −8.4 to −0.3, Cohen’s d = 0.52). This may be related to the fact that PD and HD applications are different techniques [33]. On the other hand, no significant difference was observed between PD and HD patients in terms of cardiomegaly, redistribution, interstitial pattern, pleural fluid, pleural thickness, and alveolar infiltration variables on chest radiographs taken 17 months apart (Table 4; *p* > 0.05). Furthermore, both HD and PD patients likely experienced similar rates of pulmonary redistribution due to increased pulmonary venous pressure due to fluid imbalance. Moreover, both treatment modalities cause interstitial edema and thickening of the lungs, predisposing patients to the formation of an interstitial pattern on chest radiographs. Therefore, since similar etiologic factors lead to similar chest X-ray findings, no significant difference was found between these parameters.

In our study, total dialysis times and survival rates for HD and PD methods were analyzed; no significant difference was found between them in terms of survival (Figure 2, *p* = 0.462). In a prospective cohort study conducted in 2023 with 65 PD and HD patients matched by the propensity score-matching method, patients were followed up for survival, and no significant difference was found between the two dialysis methods in terms of survival [38]. This result is consistent with our findings.

Univariate Cox regression analysis revealed that age, serum albumin, hemoglobin, ferritin, iPTH and potassium levels, and clinical status parameters had significant effects on survival (Table 5; *p* < 0.05 for all).

PTH is an 84-amino-acid protein that targets various organs such as bone, kidney, and intestine. PTH is also known as a uremic toxin; serum levels increase as CKD progresses and have many systemic side effects, including cardiovascular disease [39]. iPTH is the biologically active PTH molecule that detects full-length 1–84 bio-PTH and 7–84 PTH fragments [40]. iPTH tests accurately assess the activity of the parathyroid gland in patients with kidney disease and dialysis by measuring the active portion of the parathyroid hormone. Conventional PTH tests can overestimate results by measuring biologically inactive PTH fragments, so iPTH tests give more specific and accurate results.

It has been shown that a steady increase in PTH levels occurs as GFR starts to decrease in chronic kidney patients [41]. In patients with ESRD, PTH levels are frequently monitored to assess parathyroid function. This is necessary to manage disorders of bone and mineral metabolism.

Increased iPTH levels in dialysis patients are closely associated with secondary hyperparathyroidism. In the kidneys, 25-hydroxy vitamin D is converted to its active form, 1,25-dihydroxy vitamin D, by PTH. The resulting 1,25-dihydroxy vitamin D suppresses PTH production through a negative feedback mechanism. FGF-23 production in bone osteocytes is controlled by phosphate, 1,25-dihydroxy vitamin D, and PTH. Increased FGF-23 levels decrease PTH production by acting on the FGFR1-αKlotho receptor complex in parathyroid glands [42]. However, inhibitory feedback mechanisms in ESRD become inadequate due to the downregulation of Ca^2+^-sensitive, vitamin D, and FGF23/Klotho receptors in the parathyroid gland. In addition, end-organ ineffectiveness of PTH due to ESRD also contributes to the gradual increase in PTH synthesis and release [43]. Parathyroid glands undergo nodular hyperplasia due to negative feedback mechanisms constantly stimulating and disrupting to maintain calcium, phosphate, and vitamin D balance. Secondary hyperparathyroidism develops. Parathyroidectomy is required in severe and medically refractory patients [44,45,46]. In our study, iPTH levels were lower in the Ex group compared to the survivors (Table 5). This may be related to changes in PTH metabolism in critical illness or severe inflammation, impaired parathyroid function due to malnutrition and poor general health, and excessive suppression of iPTH due to overuse of drugs that lower iPTH levels, such as calcimimetics, vitamin D analogs, and phosphate binders. In particular, a 2021 study emphasized that using vitamin D analogs is not neutral in the development of hypercalcemia [47]. This finding suggests that vitamin D analogs may suppress PTH release, increasing the risk of hypercalcemia. Considering all these factors, it can be concluded that the lower iPTH levels in the Ex group may be due to a multifactorial effect, especially the overuse of iPTH-suppressing drugs. In a study conducted in 2017 and similar to our study, the relationship between iPTH levels of 115 HD patients within 1 year and mortality was investigated, and it was found that low iPTH levels were associated with mortality [48]. The results of our study are consistent with this literature.

Control of albumin, ferritin, hemoglobin, and potassium levels and clinical status has a positive impact on survival time. These findings emphasize that close monitoring and management of biochemical and clinical parameters are vital to improving patient prognosis.

Multivariate Cox regression analysis evaluated the independent effects of age, albumin, hemoglobin, ferritin, iPTH, and potassium levels and clinical status on survival. Age, iPTH levels, and clinical status were found to be prognostic factors affecting survival (Table 6). Increasing age was found to negatively affect survival (Table 5, *p* = 0.016; Table 6, *p* = 0.040). Studies have shown that survival of dialysis patients decreases with age due to the emergence of new comorbidities (infection, cardiovascular diseases) or deterioration of existing morbidities (hypertension, type 2 diabetes mellitus), malnutrition, decrease in physiological reserves, decrease in muscle mass and functions due to decrease in physical activities, mood disorders, difficulties in adaptation to treatment, and decrease in social life support [49,50]. Our results are consistent with this literature. It was found that a decrease in iPTH levels negatively affected survival (Table 5, *p* = 0.013; Table 6, *p* = 0.040). This result contradicts some studies in the literature. PTH levels are not a clear indicator of mortality in dialysis patients. Although only long-term studies have found that increased PTH levels are associated with mortality, the specific PTH values for this association are quite different [51,52]. Molecules that affect PTH such as calcium, phosphorus and vitamin D show a more consistent relationship with mortality. Increases in calcium levels above 10.5 mg/dL and, more importantly, increases in calcium and phosphorus and exceeding the limit value (Ca*P) of 55 mg^2^/dL^2^ increase the risk of calcification, especially in vessels and soft tissues. Due to calcified vascular structures, vascular walls harden, and their elasticity decreases, increasing the risk of mortality by increasing the development of cardiovascular events, myocardial infarction, and cerebrovascular events [53,54]. Keeping PTH levels under control at optimum levels in dialysis patients is essential for improving survival rates. However, drugs that lower iPTH levels are used to achieve this control. Over time, changes in iPTH levels can occur for reasons such as patients not following their diet. In the COSMOS study conducted in 2015, a higher iPTH increase in patients with low baseline iPTH levels was associated with lower mortality [55]. In another study conducted in 2015 and similar to ours, low iPTH levels were associated with high mortality [48]. Although both studies differ from our study in that they included only HD patients and focused on iPTH levels, our results are compatible. A large cohort study conducted in 2017 to determine mortality rates only in HD patients found that low and intermediate iPTH levels (<75 pg/mL, 75–150 pg/mL) were associated with a high mortality risk. However, high iPTH levels (300–600 pg/mL or more) did not show an increased mortality risk [56].

Our study correlated with radiologic status and clinical status (Table 8; effect size, 0.287; *p* = 0.016). This correlation suggests that evaluating both types of status together will increase survival. A Bayesian correlation test to evaluate the relationship between iPTH levels and chest X-ray findings showed a significant correlation between iPTH levels and PVPW (Table 7; mean r = −0.241, 95% CI: −0.457, −0.023). This finding suggests that there may be a relationship between iPTH levels and the width of pulmonary vascular structures.

The relationship between iPTH and PVPW was analyzed using linear regression analysis, and this relationship is shown in the scatter plot in Figure 3. The analysis revealed a weak negative correlation between iPTH and PVPW with R^2^ = 0.064. Despite the low R^2^ value, iPTH was able to explain small changes in PVPW, reflecting a statistically significant clinical association (*p* = 0.036).

This correlation suggests that the iPTH/PVPW ratio should be carefully monitored in dialysis patients. The small but significant effects of iPTH on PVPW may allow early diagnosis of possible complications in this patient group. This finding suggests that the relationship between iPTH and PVPW may play an essential role in clinical management and has the potential to be used as an additional biomarker in the monitoring of dialysis patients.

Multivariate Cox regression analysis revealed that the iPTH/PVPW ratio significantly affected mortality [Table 9, β = −0.073, *p* = 0.035, HR = 0.930, 95% CI (0.869–0.995)]. Each unit increase in this ratio increases the probability of survival by approximately 7.5%. This finding suggests that a higher iPTH/PVPW ratio is associated with longer expected survival in dialysis patients. This emphasizes the importance of the balance between bone metabolism and vascular health and may offer a potential therapeutic target. It also suggests that the iPTH/PVPW ratio may be a clinically important prognostic indicator and should be considered in predicting survival or mortality. To further explore this relationship, we evaluated the potential of the iPTH/PVPW ratio to predict mortality. ROC curve analysis revealed that the iPTH/PVPW ratio performed moderately well in predicting mortality (Figure 4, AUC = 0.606, *p* > 0.05), although it was not statistically significant, which may be due to the small size of our study group. Using ≤6.8 as the cut-off value, the sensitivity and specificity were 71.9% and 56.8%, respectively. These findings suggest mortality is higher when the iPTH/PVPW ratio is ≤6.8 (Figure 4).

The effects of parathormone on the vascular system are well-known. Increased PTH levels can trigger vascular smooth muscle cells (VSMCs) to transform into osteoblast-like cells, leading to vascular calcification. This transformation of VSMCs promotes calcium phosphate accumulation in vascular tissue, which is considered an active cell regulatory mechanism. The details of this mechanism are better understood in recent studies. In particular, high phosphate levels have been shown to promote phenotype change and osteogenic transdifferentiation of VSMCs. This leads to increased vascular calcification and associated cardiovascular morbidity and mortality [57,58].

This biological mechanism supports the findings related to the iPTH/PVPW ratio observed in our study. The effect of PTH on VSMCs and their transformation into an osteoblast-like phenotype is one of the fundamental processes underlying calcification. Therefore, the iPTH/PVPW ratio is a biologically meaningful indicator that can be used to predict vascular calcification and cardiovascular morbidity and mortality.

Increased PVPW is associated with volume overload, pulmonary artery pressure, heart failure, pulmonary edema, vascular stiffness, and elasticity. These biological mechanisms suggest that increased vascular stiffness and decreased vascular elasticity may be associated with cardiovascular complications and thus reduced survival. An iPTH/PVPW ratio of less than 6.8 suggests that vascular complications may be higher and thus associated with higher mortality rates. A lower iPTH/PVPW ratio has been linked to worse clinical outcomes, emphasizing the importance of cardiovascular health management.

In clinical practice, this ratio can be used to identify patients at high mortality risk early. In routine clinical practice, dialysis patients’ serum iPTH levels and chest radiographs are regularly monitored. PVPW is an important parameter that can be obtained from routine chest radiographs. A monitoring program integrating these parameters can improve patient care and contribute to the prevention of complications. For example, assessing the iPTH/PVPW ratio quarterly or semiannually may enable more effective management of patients’ clinical conditions.

However, more comprehensive and large-scale studies are needed to establish this relationship more precisely.

The most important limitations of our study are that it was retrospectively designed and conducted in a single center, and the sample group was small. This study has a low statistical power value due to the small sample size (70 patients total, 33 PD and 37 HD), and type 2 errors should be taken into account when evaluating the results. Although the iPTH/PVPW ratio was found to be a risk factor for mortality in our study, the results should be interpreted with caution as they have not yet been validated in an independent cohort study. Future studies should more thoroughly investigate the clinical significance and biological plausibility of the iPTH/PVPW ratio.

## 5. Conclusions

This is the first study to evaluate the effects of chest radiography and biochemical parameters on survival in PD and HD patients. It aims to contribute to a better understanding of prognostic factors in dialysis patients. Routine radiologic examinations and biochemical monitoring enable clinical follow-up of treatment efficacy. Furthermore, these findings provide information to help develop individualized treatment strategies in the follow-up of dialysis patients.

Prevention of chronic diseases such as hypertension and type 2 diabetes mellitus and effective treatment management in individuals diagnosed with these diseases play an essential role in improving survival. Ensuring infection control, regular patient education in PD patients, and regular follow-up for cardiovascular diseases, especially myocardial infarction, in HD patients is a critical factor in improving survival rates.

The results of our study revealed that the combined evaluation of chest radiography findings and biochemical parameters in managing HD and PD patients is important in improving survival rates. Another important conclusion is that uncontrolled suppression of iPTH levels may increase mortality; therefore, caution should be exercised in the use of drugs while controlling iPTH. Another important conclusion is that the calculation of iPTH/PVPW values from chest radiographs and routine biochemical parameters during routine follow-up of PD and HD patients and evaluation of iPTH/PVPW values with other clinical parameters and biomarkers in cases where they are less than 6.8 may increase survival by determining the risks of patients in terms of mortality and monitoring them more closely and carefully.

In future prospective studies and clinical applications, chest radiographs and routine biochemical parameters should be evaluated together. In the management of HD and PD patients, careful control of iPTH levels may play a critical role in increasing survival rate. iPTH/PVPW may play a critical role in determining mortality risk.

## Figures and Tables

**Table 1:** Etiologic distribution of dialysis patients.

Dialysis Etiology	n	n%
Type 1 diabetes mellitus	1	1.4%
Type 2 diabetes mellitus	19	27.1%
Hypertension	34	48.6%
Glomerulonephritis	6	8.6%
Urogenital disorders	8	11.4%
Pyelonephritis	1	1.4%
Amyloid nephropathy	1	1.4%
Total	70	100%

**Table 2:** Causes of mortality in patients undergoing PD and HD.

Ex Cause	PD (n = 33)		HD (n = 37)		*p* *
	n	%	n	%	
Pneumonia	1	7.7%	3	15.8%	0.590
Heart failure	2	15.4%	1	5.3%	0.567
Aortic dissection	1	7.7%	0	0.0%	0.410
Dialysis complications	2	15.4%	2	10.5%	1.000
Peritonitis	5	38.5%	0	0.0%	0.005
Myocardial infarction	1	7.7%	2	10.5%	1.000
Other cardiovascular causes	1	7.7%	9	47.4%	0.013
Malignancy	0	0.0%	2	10.5%	0.506
Total	13	100%	19	100%	0.008

*p* * = Fisher’s exact test; n (%).

**Table 3:** Comparison of diaphragmatic height, pulmonary artery width, pulmonary vascular pedicle width, heart size, and cardiothoracic index, biochemical parameters at baseline and after 17 months in PD and HD groups.

	Comparison of Baseline—Values at 17 Months					
	PD ^a^		HD ^a^		PD vs. HD ^b^	
	Mean (95% Confidence Interval)	Effect Size * (Cohen’s d)	Mean (95% Confidence Interval)	Effect Size * (Cohen’s d)	Mean (95% Confidence Interval)	Effect Size ** (Cohen’s d)
Diaphragm height, mm	−3.3 (−5.4–−1.2)	−0.62	1 (−2.6–4.7)	0.10	−4.4 (−8.4–−0.3)	0.52
Pulmonary artery width, mm	−0.4 (−1.2–0.4)	−0.18	−1.3 (−2.1–−0.6)	−0.63	0.9 (−0.2–1.9)	−0.42
Pulmonary vascular pedicle width, mm	−2.8 (−5.3–−0.3)	−0.43	−3.6 (−6.2–−1.1)	−0.50	0.8 (−2.7–4.3)	−0.11
Heart width, mm	−4.4 (−10.3–1.6)	−0.27	−3.7 (−7.5–0.1)	−0.34	−0.7 (−7.4–6)	0.05
Cardiothoracic index	0 (0–0)	−0.04	0 (0–0)	−0.32	0 (0–0)	−0.05
Urea, mg/dL	−1.3 (−19.6–17)	−0.03	11.8 (−4.1–27.7)	0.25	−13.1 (−36.4–10.3)	0.27
Albumin, g/dL	−0.1 (−0.2–0.1)	−0.16	−0.1 (−0.4–0.1)	−0.21	0.1 (−0.2–0.4)	−0.17
Hemoglobin, g/dL	−0.1 (−0.7–0.6)	−0.04	−0.1 (−0.8–0.6)	−0.04	0 (−0.9–0.9)	−0.01
Ferritin, µg/L	−88.9 (−155.5–−22.4)	−0.49	−107.8 (−235.1–19.4)	−0.29	18.9 (−119.9–157.7)	−0.06
Calcium, mg/dL	0.1 (−0.2–0.4)	0.12	0 (−0.4–0.4)	−0.01	0.1 (−0.4–0.6)	−0.11
Phosphate, mg/dL	0 (−0.6–0.7)	0.02	0.4 (−0.3–1.1)	0.19	−0.3 (−1.3–0.6)	0.18
iPTH, ng/dL	−237.2 (−391.2–−83.2)	−0.57	−73.3 (−182.6–36)	−0.23	−163.9 (−346.1–18.3)	0.44
Potassium, mmol/L	0.2 (−0.1–0.4)	−0.75	0.1 (−0.1–0.4)	0.04	0.1 (−0.3–0.4)	−0.07
Creatinine, mg/dL	−0.1 (−1–0.7)	−0.06	−0.7 (−1.6–0.1)	−0.31	0.6 (−0.5–1.7)	−0.26
PO_2_, mm Hg	0.2 (−7.2–7.6)	0.01	2 (−4–7.9)	0.12	−1.8 (−10.9–7.3)	0.10
PCO_2_, mm Hg	−1.2 (−3.8–1.4)	−0.17	−1.2 (−3.3–0.9)	−0.20	0 (−3.2–3.3)	0.001
SO_2_, %	1.0 (−9.3–11.3)	0.04	6.7 (0.5–12.9)	0.37	−5.7 (−17.3–5.9)	0.25

^a^ Bayesian analysis for related samples, ^b^ Bayesian analysis for independent samples. iPTH, intact parathormone; PO_2_, partial oxygen pressure; PCO_2_, partial carbon dioxide pressure; SO_2_ INTERT, oxygen saturation. * Cohen’s d = (Mean2 − Mean1)/SD_pooled_ [d = 0.20 small, d = 0.50 medium, and d = 0.80 large effect size], ** Cohen’s d = Mean_difference_/SD_difference_ [d = 0.20 small, d = 0.50 medium, and d = 0.80 large effect size].

**Table 4:** Comparison of the presence of cardiomegaly, redistribution, interstitial pattern, pleural fluid, pleural thickness, and alveolar infiltration variables at baseline and at the end of the first year in PD and HD groups.

Baseline		PD (n = 33)				HD (n = 37)		
		17 Months Later		*p* *		17 Months Later		*p* *
		No	Yes			No	Yes	
Cardiomegaly	No	14	6	0.508		11	4	0.125
	Yes	3	10			0	20	
Redistribution	No	14	1	0.219		12	2	1.000
	Yes	5	13			1	21	
Interstitial pattern	No	18	2	0.453		13	4	1.000
	Yes	5	8			5	15	
Pleural fluid	No	20	5	1.000		28	5	0.219
	Yes	5	3			1	3	
Pleural thickness	No	26	1	1.000		25	0	1.000
	Yes	0	6			0	11	
Alveolar infiltration	No	30	2	0.500		33	1	1.000
	Yes	0	1			2	1	
Atelectasis	No	30	1	1.000		37	0	
	Yes	1	1			0	0	

* McNemar.

**Table 5:** Results of univariate Cox regression analysis of the effects of possible factors on survival.

		Survived (n = 38)	Ex (n = 32)	Effect Size	Univariate Cox Regression	
					*p*	HR (95% CI)
Age, year		52.2 ± 12.7	63.5 ± 10.8	0.95 ^a^	0.016	1.034 (1.006–1.062)
Diaphragm height, mm		21.6 ± 11.4	23.5 ± 16.4	0.14 ^a^	0.599	1.007 (0.980–1.036)
Pulmonary artery diameter, mm		14.5 ± 2.8	15.3 ± 2.8	0.32 ^a^	0.302	1.070 (0.941–1.218)
Pulmonary vascular pedicle width, mm		56.5 ± 11.9	57 ± 10.2	0.05 ^a^	0.140	1.024 (0.992–1.056)
Width of the heart, mm		147.8 ± 23.1	152.8 ± 26.1	0.20 ^a^	0.156	1.011 (0.996–1.025)
CTI		0.5 ± 0.1	0.5 ± 0.1	0.23 ^a^	0.478	5.169 (0.055–484.6)
Urea, mg/dL		129.3 ± 43.4	128.7 ± 34.1	−0.01 ^a^	0.831	1.001 (0.991–1.011)
Albumin, g/dL		3.7 ± 0.4	3.4 ± 0.6	−0.72 ^a^	0.005	0.339 (0.159–0.726)
Hemoglobin, g/dL		11.1 ± 2	10.4 ± 1.9	−0.33 ^a^	0.040	0.810 (0.662–0.990)
Ferritin, µg/L		229.7 ± 180.1	394.6 ± 334.3	0.63 ^a^	0.047	1.001 (1.000–1.002)
Calcium, mg/dL		9.1 ± 0.8	9 ± 0.8	−0.05 ^a^	0.546	0.878 (0.575–1.340)
Phosphate, mg/dL		5.4 ± 1.6	5.5 ± 1.4	0.05 ^a^	0.650	1.059 (0.826–1.359)
iPTH, ng/dL		524.8 ± 380.1	388 ± 321.3	−0.39 ^a^	0.013	0.998 (0.997–1.000)
Potassium, mmol/L		4.9 ± 0.7	4.7 ± 0.8	−0.33 ^a^	0.035	0.630 (0.410–0.968)
Creatinine, mg/dL		7.4 ± 3.3	8.2 ± 2.6	0.26 ^a^	0.505	0.957 (0.842–1.088)
PO_2_, mm Hg		47.3 ± 13.4	49.4 ± 16.1	0.14 ^a^	0.955	0.999 (0.976–1.023)
PCO_2_, mm Hg		39.8 ± 5.7	39.8 ± 7.7	−0.01 ^a^	0.919	1.003 (0.949–1.060)
SO_2_, %		74.5 ± 16.3	76.2 ± 19	0.10 ^a^	0.793	1.003 (0.982–1.024)
Charlson Comorbidity Index		3.5 ± 1.7	4.5 ± 1.2	0.66 ^a^	0.086	1.208 (0.973–1.499)
Gender	Female	26 (68.4)	18 (56.3)	0.13 ^b^		1 (reference)
	Male	12 (31.6)	14 (43.8)		0.159	1.665 (0.819–3.387)
Dialysis type	PD	20 (52.6)	13 (40.6)	0.12 ^b^		1 (reference)
	HD	18 (47.4)	19 (59.4)		0.481	1.293 (0.633–2.642)
Cardiomegaly	No	23 (60.5)	13 (40.6)	0.20 ^b^		1 (reference)
	Yes	15 (39.5)	19 (59.4)		0.154	1.693 (0.820–3.492)
Aortic calcification	No	13 (34.2)	3 (9.4)	0.30 ^b^		1 (reference)
	Yes	25 (65.8)	29 (90.6)		0.252	2.010 (0.608–6.639)
Redistribution	No	14 (36.8)	15 (46.9)	−0.10 ^b^		1 (reference)
	Yes	24 (63.2)	17 (53.1)		0.680	1.166 (0.563–2.415)
Interstitial pattern	No	20 (52.6)	17 (53.1)	−0.01 ^b^		1 (reference)
	Yes	18 (47.4)	15 (46.9)		0.714	1.143 (0.559–2.338)
Pleural fluid	No	29 (76.3)	29 (90.6)	−0.20 ^b^		1 (reference)
	Yes	9 (23.7)	3 (9.4)		0.362	0.572 (0.173–1.898)
Pleural thickness	No	30 (78.9)	22 (71)	0.09 ^b^		1 (reference)
	Yes	8 (21.1)	9 (29)		0.624	0.812 (0.354–1.865)
Alveolar infiltration	No	36 (94.7)	30 (93.8)	0.02 ^b^		1 (reference)
	Yes	2 (5.3)	2 (6.3)		0.530	1.587 (0.376–6.709)
Atelectasis	No	37 (97.4)	31 (96.9)	0.02 ^b^		1 (reference)
	Yes	1 (2.6)	1 (3.1)		0.910	1.123 (0.152–8.321)
Radiological status	Deterioration	7 (18.4)	17 (53.1)	−0.36 ^b^		1 (reference)
	Stable or improved	31 (81.6)	15 (46.9)		0.055	0.500 (0.246–1.015)
Family history	No	28 (73.7)	24 (75)	−0.02 ^b^		1 (reference)
	Yes	10 (26.3)	8 (25)		0.730	0.866 (0.382–1.961)
Clinical status	Deterioration	15 (39.5)	30 (93.8)	−0.56 ^b^		1 (reference)
	Stable or improved	23 (60.5)	2 (6.3)		0.003	0.118 (0.028–0.495)

Mean ± standard deviation, n (%); CTI, cardiothoracic index; iPTH, intact parathormone; PO_2_, partial oxygen pressure; PCO_2_, partial carbon dioxide pressure; SO_2_, oxygen saturation. ^a^ $Cohen^{’}s\;d=(Mean2-Mean1)/{SD}_{pooled}$ [d = 0.20 small, d = 0.50 medium, and d = 0.80 large effect size]. ^b^ $w=\sqrt{X^{2}/N}$ [w = 0.10 small, w = 0.30 medium, and w = 0.50 large effect size].

**Table 6:** Results of multivariate Cox regression analysis of the effects of possible factors on survival.

	Beta	*p*	Hazard Ratio (95% Confidence Interval)
Age, year	0.044	0.007	1.045 (1.012–1.078)
Albumin, g/dL	−0.501	0.213	0.606 (0.275–1.334)
Hemoglobin, g/dL	−0.166	0.129	0.847 (0.683–1.050)
Ferritin, µg/L	0.001	0.255	1.001 (1.000–1.001)
iPTH, ng/dL	−0.002	0.040	0.998 (0.997–1.000)
Potassium, mmol/L	−0.455	0.071	0.635 (0.387–1.040)
Clinical status	−1.933	0.009	0.145 (0.034–0.621)

iPTH, intact parathormone.

**Table 7:** Bayesian correlation test results between iPTH level and chest X-ray findings.

			Diaphragm Height	Pulmonary Artery Diameter	PVPW	CTI
iPTH	Mean r		−0.036	−0.128	−0.241	−0.071
	95% Confidence interval	Lower	−0.265	−0.352	−0.457	−0.299
		Upper	0.194	0.100	−0.023	0.156

r, Bayesian correlation coefficient; CTI, cardiothoracic index; iPTH, intact parathormone; PVPW, pulmonary vascular pedicle width.

**Table 8:** Chi-square test results of the relationship between radiological and clinical status in PD and HD patients.

			Clinical Status		Effect Size	*p*
			Deterioration	Stable or Improved		
PD and HD	Radiological status	Deterioration	20	4	0.287	0.016
		Stable or improved	25	21		

**Table 9:** Multivariate Cox regression analysis results of the effect of iPTH/PVPW on mortality.

	Beta	*p*	Hazard Ratio (95% Confidence Interval)
Age, year	0.040	0.016	1.040 (1.008–1.074)
iPTH/PVPW	−0.073	0.035	0.930 (0.869–0.995)
Albumin, g/dL	−0.500	0.214	0.606 (0.275–1.335)
Hemoglobin, g/dL	−0.160	0.141	0.852 (0.689–1.054)
Ferritin, µg/L	0.000	0.354	1.000 (1.000–1.001)
Potassium, mmol/L	−0.440	0.083	0.644 (0.391–1.059)
Clinical status	−1.985	0.008	0.137 (0.032–0.589)

iPTH, intact parathormone; PVPW, pulmonary vascular pedicle width.

## Data Availability

Our study data contain patients’ personal information and, therefore, are unavailable for sharing due to the ‘Personal Data Protection Law’ and ethical reasons.
